# Molecular Subtyping and Prognostic Prediction in Pancreatic Cancer Based on Mitophagy-Related Genes

**DOI:** 10.7150/ijms.121350

**Published:** 2026-01-14

**Authors:** Yunlong Cai, Taohua Yue, Yongchen Ma, Guanyi Liu, Jixin Zhang, Long Rong

**Affiliations:** 1 Endoscopy Center, Peking University First Hospital, Beijing 100034, China.; 2 Pathology Department, Peking University First Hospital, Beijing 100034, China.

**Keywords:** pancreatic cancer, mitophagy, prognosis, gene expression analysis

## Abstract

**Background:** Pancreatic cancer (PaC) is characterized by poor prognosis. This study aimed to identify mitophagy-related clusters and develop a prognostic model for PaC.

**Methods:** Differentially expressed genes (DEGs) between PaC and normal tissues were identified from the TCGA and GTEx cohorts. Mitophagy-related genes (MRGs) were sourced from Reactome, GO, and KEGG databases. The intersection of DEGs and MRGs identified differentially expressed MRGs (DeMRGs). Consensus clustering identified PaC subtypes based on DeMRG expression. Univariate Cox analysis was used to find prognosis-related genes, and LASSO regression analysis was employed to develop the prognostic model. A nomogram was constructed to predict survival probabilities.

**Results:** A total of 7,240 DEGs were identified between PaC tissues and normal controls. From these, 12 DeMRGs were identified, and consensus clustering revealed three distinct molecular clusters. A prognostic model based on six significant genes (PAPPA, NBPF12, CXCL11, CKLF-CMTM1, CCDC6, AHNAK) was developed using LASSO regression analysis. This model demonstrated good predictive performance for overall survival in the TCGA cohort, with AUC values of 0.78, 0.74, and 0.82 for 1-, 2-, and 3-year survival in the training set, and 0.73, 0.82, and 0.73 in the validation set. External validation in independent GEO cohorts demonstrated moderate predictive performance. The nomogram demonstrated good calibration and accuracy in predicting survival. Significant correlations were found between the risk model and immune cell infiltration. High-risk patients showed higher sensitivity to dasatinib and staurosporine.

**Conclusions:** The study identified mitophagy-related molecular clusters and developed a prognostic model for PaC. This model may help predict overall survival and guide personalized treatment strategies for PaC patients.

## Introduction

Pancreatic cancer (PaC) is a highly lethal malignancy originating in the tissues of the pancreas, characterized by a poor prognosis, with a 5-year relative survival rate of 12% for diagnoses made between 2012 and 2018 in the United States^1^. Despite being the sixth leading cause of cancer-related deaths worldwide^2^, PaC often evades early detection due to the absence of distinctive clinical symptoms and its aggressive nature^3^. The current therapeutic approaches, primarily systemic chemotherapy and surgical resection, are limited and largely ineffective, contributing to high mortality rates^4^. These challenges underscore the critical need for novel prognostic markers and tailored treatment strategies to improve clinical outcomes for PaC patients.

Mitophagy, a selective form of autophagy targeting mitochondria for degradation, operates via ubiquitin-dependent (e.g., PINK1-PRKN pathway) and ubiquitin-independent (e.g., BNIP3L/NIX, FUNDC1) mechanisms, playing a crucial role in maintaining mitochondrial quality and cellular homeostasis^5^. In PaC, mitophagy exhibits a dual role in tumorigenesis and treatment response. The PINK1/PRKN pathway inhibits tumorigenesis by reducing inflammation and immune suppression. Conversely, the BNIP3L pathway promotes tumorigenesis by enhancing antioxidant capacity and metabolic adaptation. Therapeutically, mitophagy influences chemoresistance and sensitivity to metformin in PaC stem cells, underscoring its significance in treatment strategies^6^. Recent studies highlight the pivotal role of mitophagy-related genes (MRGs) in predicting PaC prognosis and identifying gene signatures and subtypes associated with survival outcomes, immune activity, and chemotherapy response^7^, ^8^. However, these models are limited by heterogeneity and require validation in diverse cohorts to develop reliable predictive tools for personalized treatment based on mitophagy-related molecular clusters.

In this study, we identified differentially expressed MRGs (DeMRGs) in PaC using data from The Cancer Genome Atlas (TCGA) and Genotype-Tissue Expression (GTEx) cohorts. We used consensus clustering to define distinct molecular subtypes of PaC based on DeMRG expression. A prognostic model based on DEGs between these subtypes was developed to predict overall survival (OS). The constructed nomogram was validated for accuracy and clinical utility. Our findings may enhance the understanding of PaC biology and contribute to more effective and individualized treatment approaches.

## Materials and Methods

### Study cohorts and data sources

The raw count data for the TCGA PaC cohort (including cancer and adjacent normal tissues) and GTEx normal pancreatic tissues were downloaded from the UCSC Xena Browser (https://xenabrowser.net/). The raw counts were converted to TPM expression values using the "count2tpm" function from the R package "IOBR". When a gene had multiple expression values, the median was used as the expression value. The processed expression profiles from the TCGA cohort and the GTEx cohort were then merged, and batch effects were removed using the "ComBat" function from the "sva" package. PaC cohorts GSE71729 and GSE21501 were downloaded from the Gene Expression Omnibus database (GEO, https://www.ncbi.nlm.nih.gov/geo/) for validation purposes. OS time was recorded in days. Sample information is summarized in **Table 1**. A total of 61 mitophagy-related genes (MRGs) were identified using the keyword "mitophagy" in the Reactome, Gene Ontology (GO), and Kyoto Encyclopedia of Genes and Genomes (KEGG) databases^9^-^11^.

### Identification of DeMRGs

Differentially expressed genes (DEGs) between PaC and normal controls were identified in the merged TCGA and GTEx cohorts using the R package "limma"^12^. Genes with adj.P.Val < 0.05 and log2 |fold change (FC)| > 1 were considered DEGs^13^. The intersection of DEGs and MRGs identified by a Venn diagram was identified as DeMRGs.

### Consensus clustering

PaC molecular subtypes were identified based on the expression of DeMRGs in TCGA patients with the R package "ConsensusClusterPlus"^14^. Clustering was performed 500 times using 80% of the samples per iteration. Euclidean distance was employed as the metric (reps=500, pItem=0.8, distance="euclidean"). The optimal number of subtypes (k) was determined based on tendencies and smoothness of the cumulative distribution function (CDF) curve, consistency scores, and the consensus matrix. The R packages "Rtsne" and principal component analysis (PCA) were utilized to visualize the distribution of different subtypes. Differential expression analysis between subtypes was conducted using the R package "limma", with DEGs identified by the criteria |logFC| > 1 and adj.P.Val < 0.05. The intersecting DEGs between subtypes underwent subsequent analyses.

### Functional enrichment analysis

Function enrichment of the DEGs was conducted through GO and KEGG analyses using the R package "clusterProfiler"^15^. P-values were adjusted using the Benjamini-Hochberg method, and the enrichment results with adjusted p-values were presented.

### Construction of a prognostic model

Prognosis-related genes (p < 0.05) were identified among the DEGs between PaC subtypes using a univariate COX analysis with survival data from the TCGA cohort. Patients from the TCGA cohort were then randomly divided into a training set and a validation set in a 7:3 ratio. LASSO regression analysis was conducted on the expression profiles of the prognosis-related genes in the training set using the R package "glmnet"^16^. The penalty parameter λ that optimizes the minimum criterion was determined, and genes with non-zero coefficients were identified as the model genes. LASSO was selected because it simultaneously performs variable selection and regularization, thereby reducing overfitting and enhancing model interpretability^17^. The weight coefficients were integrated with the expression levels of the model genes to calculate the risk score using the following formula:

RiskScore = 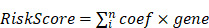


### Protein-protein interaction (PPI) network

A PPI network was constructed for the model genes to identify proteins that potentially exhibit shared functions using GeneMANIA^18^ (http://www.genemania.org).

### Construction and evaluation of a nomogram

Cox regression analysis was conducted on multiple risk factors, including risk score, age, sex, stage, and other clinical information. The results were visualized using forest plots generated by the R package "forestplot". A nomogram was constructed based on prognostic factors using the R package "rms". Its clinical decision-making performance was assessed through calibration curves produced by the "calibrate" function in the R package "rms".

### Immunotherapy response prediction

The response of the TCGA cohort to anti-PD-1/PD-L1 and anti-CTLA4 treatments was predicted using the Tumor Immune Dysfunction and Exclusion (TIDE) tool (http://tide.dfci.harvard.edu).

### Immune cell infiltration analysis

The abundance of 21 types of immune cells in TCGA patient samples was determined using the "CIBERSORT" algorithm^19^. Samples with a p-value less than 0.05 were selected for further analysis. Immune cells with zero abundance across all samples were excluded. The infiltration of the remaining immune cells was examined in patients stratified by different risk levels.

### Gene set enrichment analysis (GSEA)

The TCGA cohort was divided into high-risk and low-risk groups based on the median risk score. A differential gene expression analysis was performed on all genes between the two groups and ranked accordingly. A GSEA analysis was then conducted based on the ranking of all genes using the R package "clusterProfiler". Pathway enrichment was specifically investigated in high-risk patient cohorts, with a p.adjust value of less than 0.05.

### Drug sensitivity prediction

The "calcPhenotype" function in the "oncoPredict" package was used to analyze data from the Genomics of Drug Sensitivity in Cancer (GDSC2) and CTRP V2 databases. The expression matrix and drug treatment data from the training set were utilized to analyze the TCGA cohort. A lower predicted drug response score (PreScore) indicates a higher sensitivity to the drug.

### Statistical analysis

Statistical analysis was conducted using R (v4.3.0). Survival analysis was performed using "survival" and visualized using "survminer". ROC analysis was conducted using "timeROC" and presented using "pROC". Heatmaps were generated using "pheatmap". Result diagrams were generated using "ggplot2" or "plot". Pearson's method was employed for correlation analysis, and the t-test was utilized for comparing differences between two groups. A p-value less than 0.05 was considered statistically significant.

## Results

### Identification and functional enrichment analysis of DeMRGs

**Figure 1:**
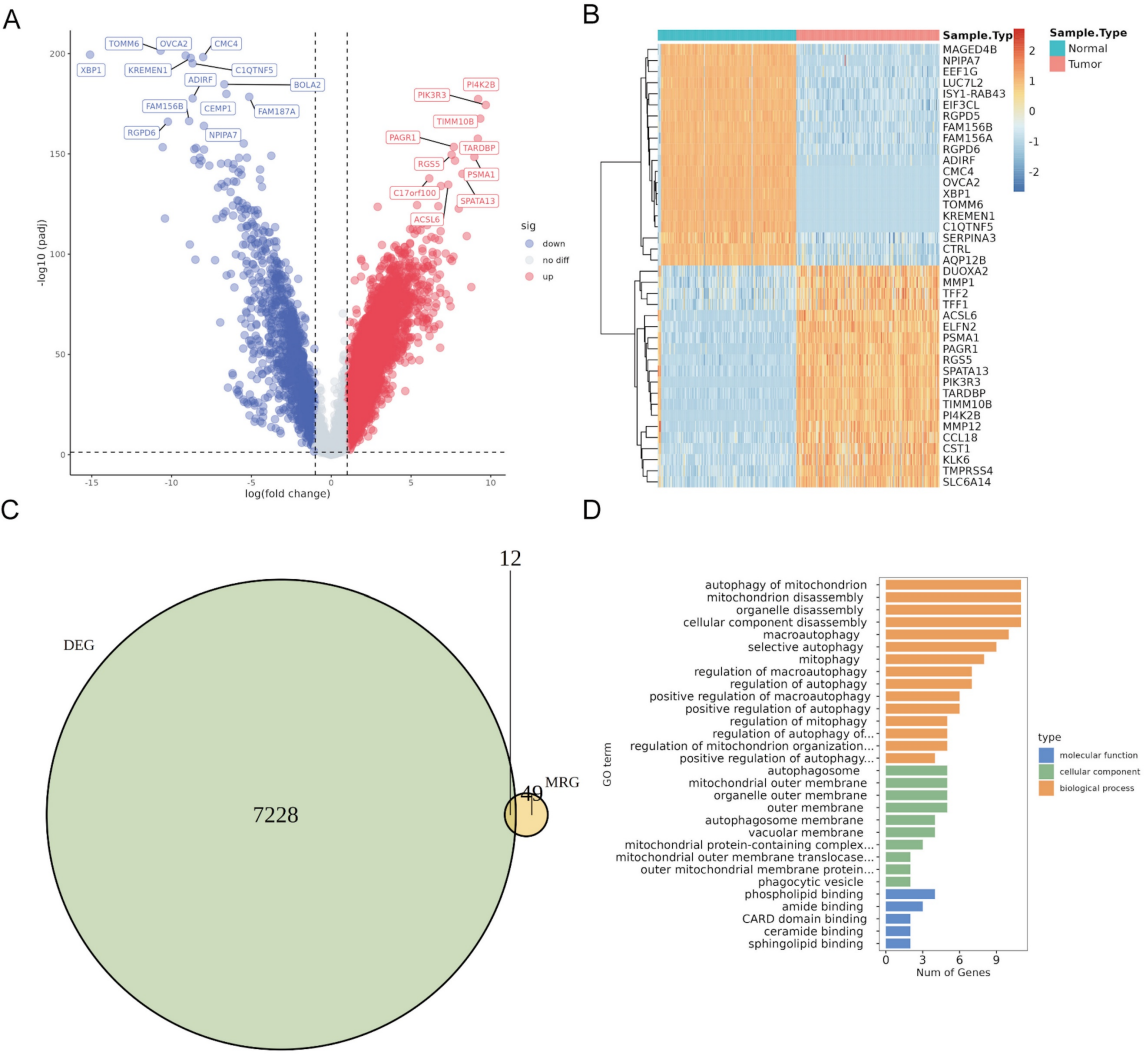
** Identification and characterization of differentially expressed mitophagy-related genes (DeMRGs) in pancreatic cancer (PaC).** (A) A volcano plot illustrates the differentially expressed genes (DEGs) from the merged TCGA and GTEx cohorts. Red dots represent upregulated genes, and blue dots represent downregulated genes. The top 20 genes with the most significant changes in expression levels are labeled. (B) A heatmap displays the top 40 DEGs. Each row represents a gene, and each column represents a sample. The color gradient indicates the level of gene expression, with red representing higher expression and blue representing lower expression. (C) A Venn diagram depicts the intersection of DEGs and MRGs, identifying 12 DeMRGs. (D) Gene Ontology (GO) functional enrichment analysis of the DeMRGs. The bar graph categorizes the enriched GO terms into biological processes (orange), cellular components (green), and molecular functions (blue). The length of each bar represents the number of genes associated with each GO term.

Differential expression analysis using the merged TCGA and GTEx cohorts revealed 7,240 DEGs (5,704 upregulated and 1,536 downregulated) in PaC tissues compared to normal controls **(Fig. 1A, Table S1)**. The top 40 DEGs with the most significant changes in expression levels were displayed in the heatmap **(Fig. 1B)**. The intersection of 61 MRGs and 7,240 DEGs identified 12 DeMRGs **(Fig. 1C)**. GO functional enrichment analysis revealed that the major enriched biological processes consisted of 15 categories, including "autophagy of mitochondrion", " mitochondrion disassembly", and "organelle disassembly". The enriched cellular components comprised five categories, such as "autophagosome", " mitochondrial outer membrane", and "organelle outer membrane". The molecular functions included six categories, such as "phospholipid binding", amide binding", and "CARD domain binding" **(Fig. 1D, Table S2)**.

### Identification of PaC subtypes based on DeMRGs

**Figure 2:**
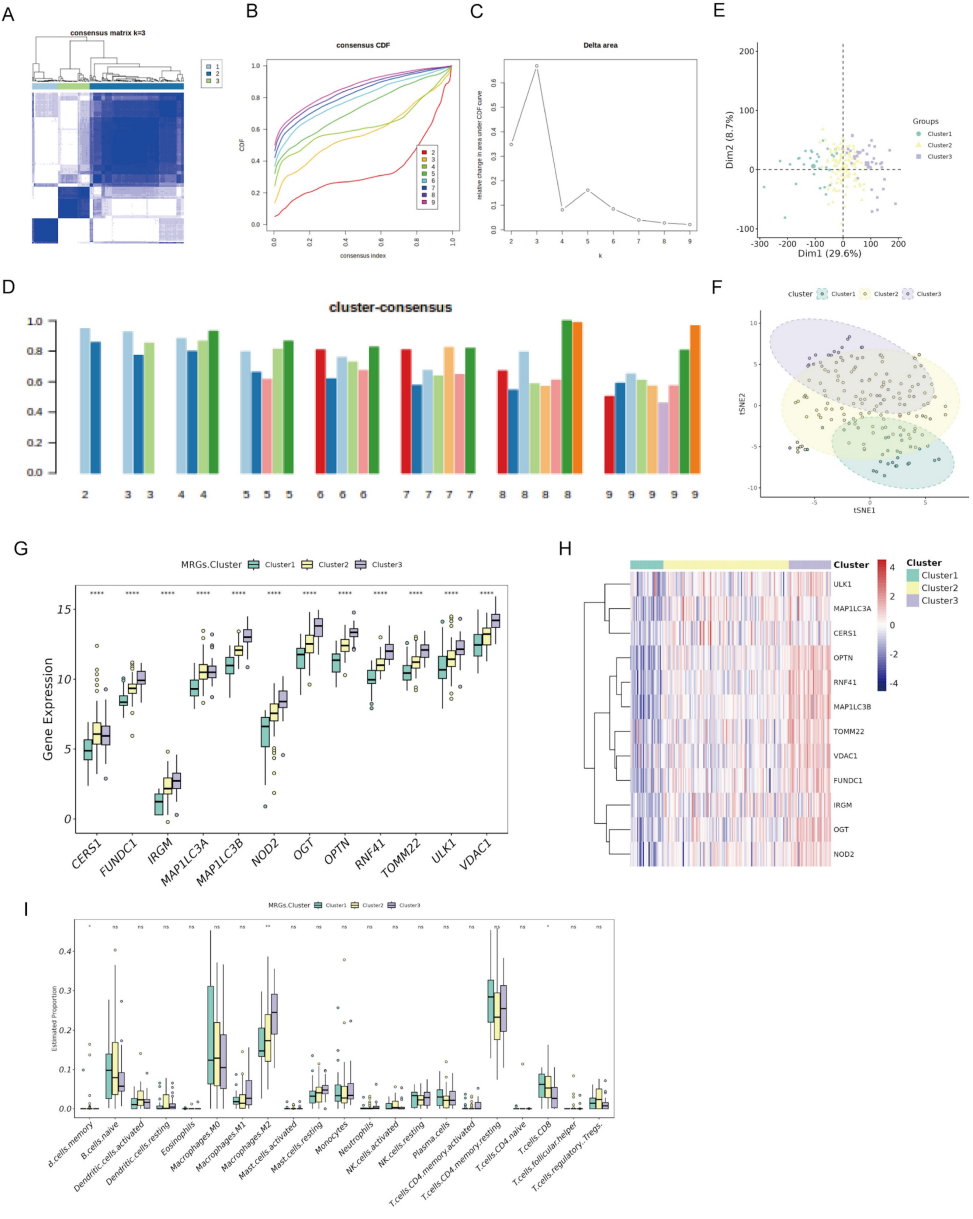
** Consensus clustering of PaC patients based on DeMRGs.** (A) Consensus matrix for k=3, showing distinct clustering into three groups with high consensus scores. (B) A consensus cumulative distribution function (CDF) plot depicts the cumulative distribution for different values of k ranging from 2 to 9. (C) A delta area plot shows the relative change in area under the CDF curve for different values of k, with a notable decrease indicating the stability of the clustering at k=3. (D) A cluster-consensus bar plot indicates the clustering consistency for each cluster. (E) A principal component analysis (PCA) scatter plot shows the distribution of the three identified clusters. (F) A t-SNE plot illustrates the clustering of the PaC patients into three distinct subtypes. Each point represents a patient and is colored according to the assigned cluster. (G, H) A box plot (G) and heatmap (H) illustrate the expression patterns of 12 DeMRGs across the identified subtypes. (I) A box plot shows differences in the abundance of 21 immune cell types among the three clusters. ^*^p < 0.05, ^**^p < 0.01, ns, non-significant.

To identify DeMRG-related PaC subtypes, we conducted consensus clustering on 177 PaC patients in the TCGA cohort using the expression profiles of 12 DeMRGs. The consensus matrix heatmap revealed three distinct clusters with high consensus scores, indicating a clear separation between the groups **(Fig. 2A)**. The CDF plot **(Figure 2B)** and the delta area plot **(Fig. 2C)** supported the stability and optimality of clustering at k = 3. The cluster-consensus bar plot demonstrated uniform clustering consistency across different clusters **(Fig. 2D)**. PCA **(Fig. 2E)** and t-SNE **(Fig. 2F)** analyses further validated the distinct separation between the three subtypes. The bar graph **(Fig. 2G)** and heatmap **(Fig. 2H)** showed significant variations in expression of the 12 DeMRGs across different subtypes. Examination of the immune microenvironment revealed notable differences in the abundance of three immune cell types, including memory B cells, M2 macrophages, and CD8 T cells **(Fig. 2I)**.

### Identification and enrichment analysis of DEGs among PaC subtypes

**Figure 3:**
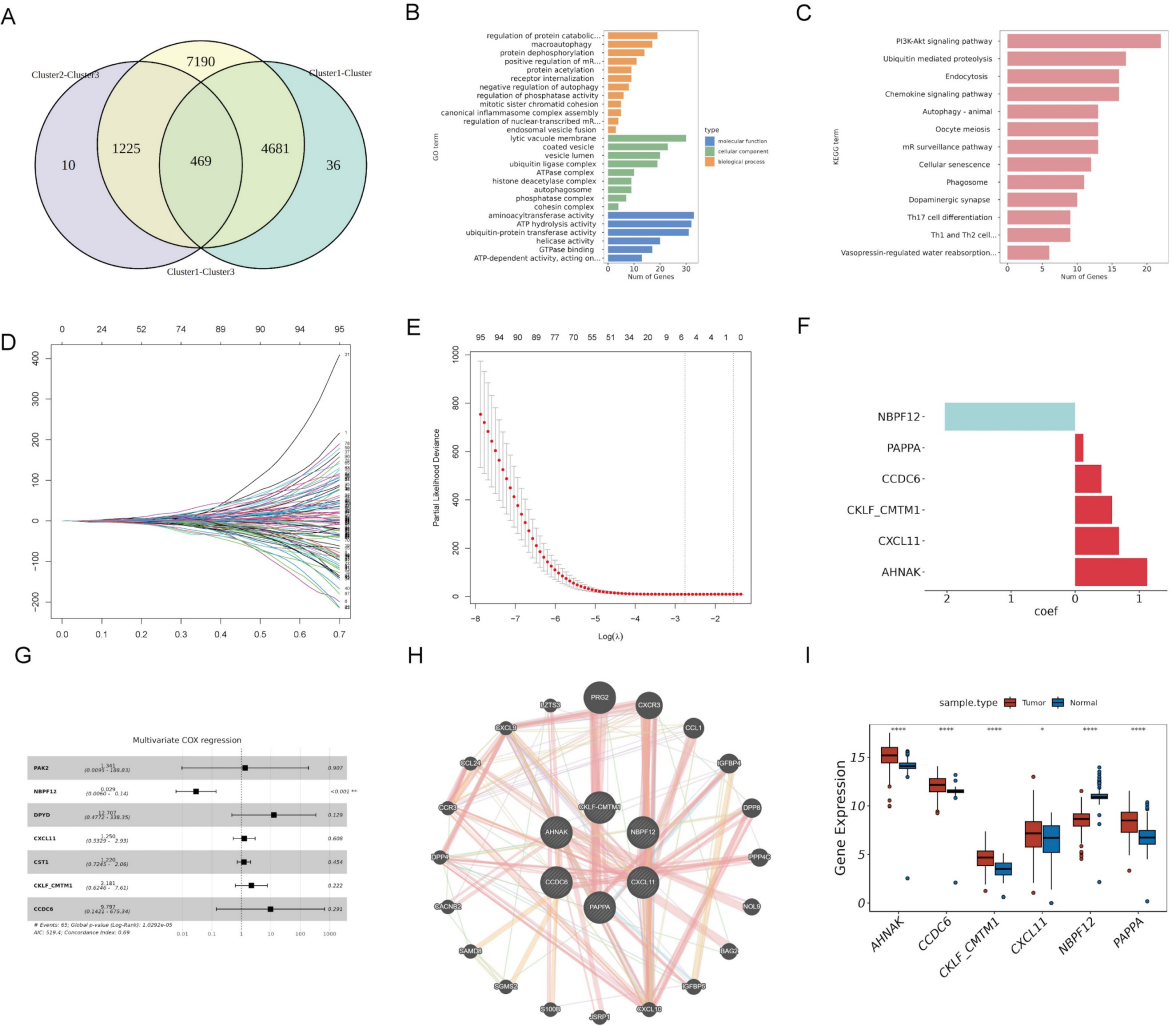
** Identification and prognostic analysis of DEGs in PaC subtypes**. (A) A Venn diagram identified 469 DEGs common across three PaC subtypes. (B, C) GO (B) and KEGG (C) functional enrichment analyses of the 469 DEGs. (D) A trace plot for the LASSO COX regression shows the coefficient paths for each variable as a function of the regularization parameter lambda. (E) Ten-fold cross-validation curve for LASSO COX regression. Dashed lines indicate minimum lambda and optimal lambda. (F) Bar plot of the weight coefficients of the six selected genes. (G) Forest plot of the multivariate COX regression analysis for the model genes. Hazard ratios (HR) and 95% confidence intervals (CI) are shown. (H) Protein-protein interaction network of the model genes and other related proteins. Nodes represent proteins, and edges represent interactions. (I) Box plots comparing the expression of levels of the model genes between PaC tissues and normal tissues.^ *^p < 0.05, ^****^p < 0.0001.

We identified 469 DEGs common across three PaC subtypes by Venn diagram analysis of genes exhibiting differential expression in cancerous versus adjacent non-cancerous tissues **(Fig. 3A)**. GO functional enrichment analysis **(Fig. 3B, Table S3)** revealed enrichment in 12 biological processes, including "macroautophagy", "protein dephosphorylation, " and "endosomal vesicle fusion". The analysis also identified enrichment in 9 cellular components, such as "the ATPase complex", "autophagosome", and "phosphatase complex". Additionally, 6 molecular functions were enriched, including "ATP hydrolysis activity", "ubiquitin-protein transferase activity", and "GTPase binding". KEGG pathway analysis **(Fig. 3C, Table S4)** showed enrichment in 13 pathways, including the "PI3K-Akt signaling pathway", "oocyte meiosis", and "phagosome".

### Construction of a prognostic risk model based on DEGs among PaC subtypes

To identify model genes among the 469 DEGs, we conducted univariate COX analysis in the TCGA cohort and obtained 101 potential prognostic genes **(Table S5)**. We divided the TCGA cohort into a training set and a validation set in a 7:3 ratio. LASSO COX analysis was then performed on the training set, and six genes (PAPPA, NBPF12, CXCL11, CKLF-CMTM1, CCDC6, and AHNAK) were selected based on the model's minimum lambda **(Fig. 3D and 3E)**. The weight coefficients of these genes are shown in **Fig. 3F**. The risk score was calculated using the formula: RiskScore = (0.129) × PAPPA + (-2.035) × NBPF12+ (0.684) × CXCL11 + (0.575) × CKLF-CMTM1 + (0.411) × CCDC6 + (1.125) × AHNAK. Subsequently, a multivariate COX analysis was conducted on these genes **(Fig. 3G)**, and a PPI network was constructed **(Fig. 3H)**. NBPF12 exhibited significant downregulation in cancer tissues compared to adjacent normal tissues, while the other five model genes exhibited significant upregulation **(Fig. 3I)**.

### Prognostic evaluation of the risk model in training and validation sets of the TCGA cohort

**Figure 4:**
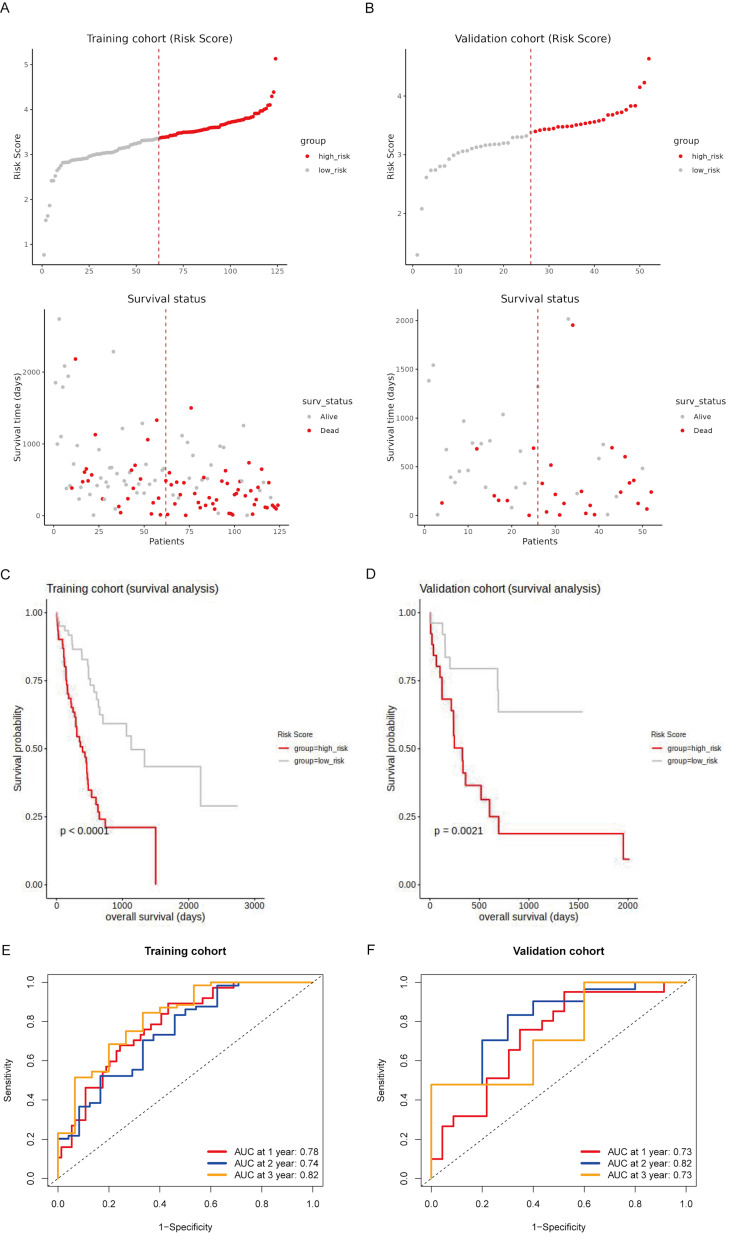
** Prognostic evaluation of the risk model in the TCGA cohort.** (A, B) Risk score distribution and survival status of patients in the training (A) and validation (B) sets. The top panel shows patients stratified into high-risk (red) and low-risk (gray) groups based on their risk scores. The bottom panel illustrates the survival status of patients, with red dots representing deceased patients and gray dots representing surviving patients. (C, D) Kaplan-Meier survival curves for the training (C) and validation (D) cohorts, comparing overall survival (OS) between high-risk (red line) and low-risk (gray line) patients. (E, F) Time-dependent ROC curves for the training (E) and validation (F) sets at 1 year, 2 years, and 3 years.

To evaluate the prognostic value of the risk model, we conducted survival analyses on both the training and validation sets of the TCGA cohort. Patients were stratified into high-risk and low-risk groups based on their risk scores, with high-risk patients showing a higher prevalence of death **(Fig. 4A and 4B)**. Kaplan-Meier survival curves confirmed that high-risk patients had significantly worse OS in both the training set (p < 0.0001; **Fig. 4C**) and the validation set (p = 0.0021; **Fig. 4D**). The time-dependent ROC analysis demonstrated high predictive accuracy in the training set, with AUCs of 0.78, 0.74, and 0.82 for 1-year, 2-year, and 3-year survival, respectively **(Fig. 4E)**. In the validation set, the AUC values were 0.73, 0.82, and 0.73 for 1-year, 2-year, and 3-year survival, respectively, indicating consistent predictive accuracy **(Fig. 4F)**. These findings suggest that the risk model effectively stratifies patients by prognosis and demonstrates reliable predictive accuracy in both the training and validation sets.

Additionally, the t-SNE plot demonstrated a clear separation among the three clusters **(Fig. S1A)**. Cluster 3 exhibited significantly higher risk scores compared to Cluster 1 (^**^p < 0.01) and Cluster 2 (^*^p < 0.05; **Fig. S1B**), suggesting that patients in Cluster 3 are at higher risk. Furthermore, a Sankey diagram illustrated the distribution of patients from each cluster into high-risk and low-risk groups and their corresponding survival status **(Fig. S1C)**. Most patients in Cluster 3 were classified as high-risk and had a higher proportion of deaths, while patients in Clusters 1 and 2 were predominantly low-risk and had better survival outcomes. This analysis reinforces the prognostic significance of the clustering.

### Validation of the prognostic value of the risk model in independent datasets and diverse clinical subgroups

To validate the prognostic value of the risk model, we conducted survival analyses using two independent datasets. Patients were stratified into high-risk and low-risk groups based on their risk scores. The survival status plot indicated that high-risk patients had a higher incidence of death **(Fig. S2A and S2B)**. Kaplan-Meier survival analysis confirmed that high-risk patients had significantly worse OS compared to low-risk patients (p = 0.01 for GSE21501, p = 0.04 for GSE71729; **Fig. S2C and S2D**). The time-dependent ROC analysis indicated moderate predictive accuracy at various time points, with AUC values at 1-year, 2-year, and 3-year being 0.66, 0.54, and 0.62 for GSE21501, and 0.59, 0.57, and 0.65 for GSE71729 **(Fig. S2E and S2F)**. These results provide supportive external evidence for the prognostic stratification ability of the risk model.

To further validate the risk model, we conducted survival analyses across different clinical and demographic subgroups. High-risk patients consistently had worse OS compared to low-risk patients in those aged ≤ 65, aged > 65, females, males, well-differentiated tumors (G1-G2), poorly-differentiated tumors (G3-G4), early-stage disease (I-II), smaller tumors (T1-T2), larger tumors (T3-T4), no metastasis (M0), no lymph node involvement (N0), and lymph node involvement (N1-N3) (all p < 0.05), except for advanced-stage disease (III-IV) and metastasis (M1) (both p > 0.05) **(Fig. S3A-H and S4A-F)**. Boxplots **(Fig. S4G)** showed that high-risk scores were associated with higher tumor grades, advanced stages, and lymph node involvement statuses.

### Construction and evaluation of nomogram

**Figure 5:**
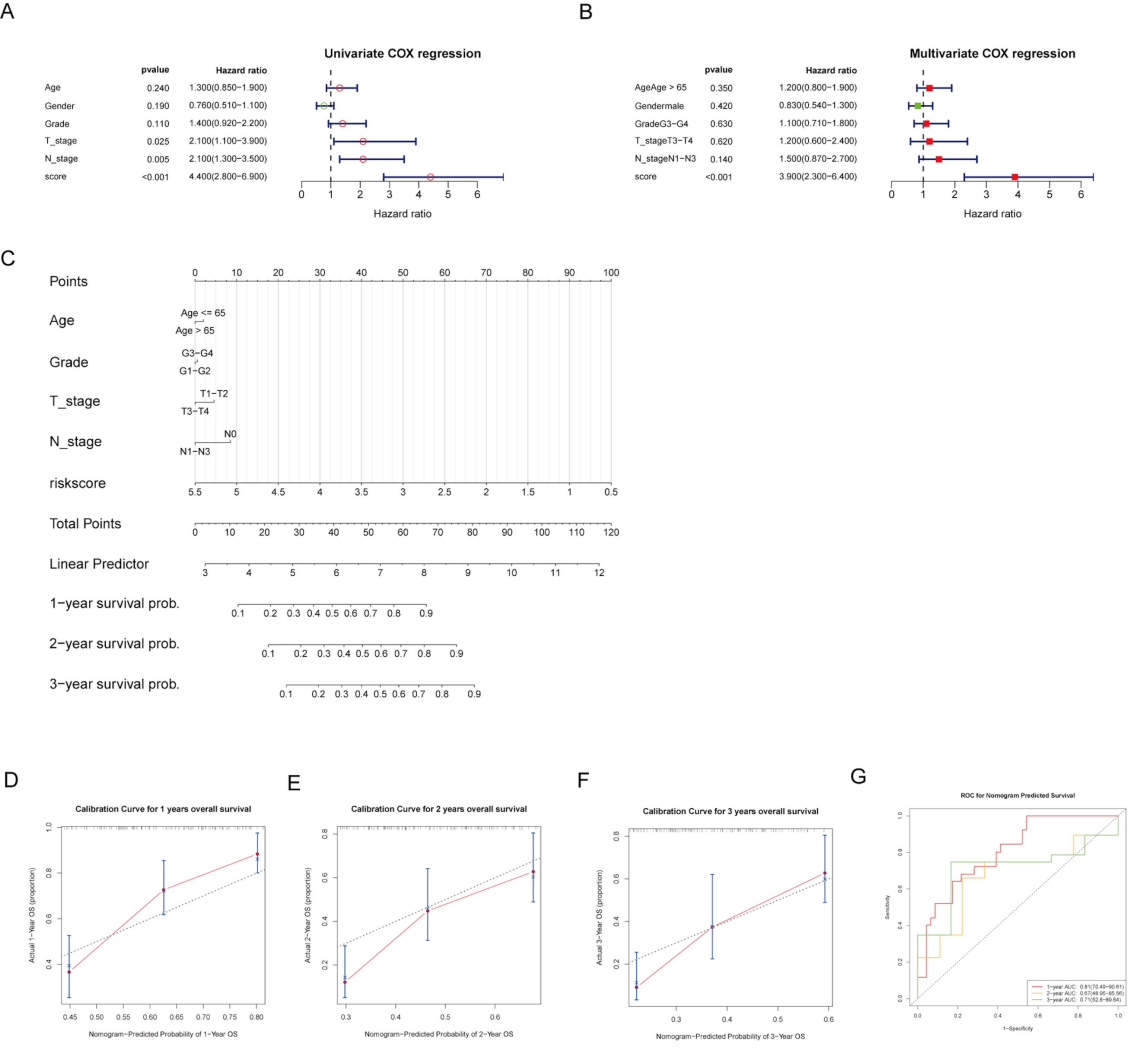
** Nomogram construction and evaluation.** (A, B) Forest plots of univariate and multivariate COX analyses for risk score and clinical factors. (C) A nomogram was developed to provide individualized survival predictions, integrating age, grade, T stage, N stage, and the risk score for 1-, 2-, and 3-year OS. (D-F) The calibration curve for 1-year OS demonstrates good agreement between predicted and observed outcomes. (G) The ROC curves for nomogram-predicted 1-, 2-, and 3-year survival.

To provide individualized survival predictions, we developed a nomogram that integrates multiple clinical variables and the risk score. The univariate Cox regression analysis revealed that risk score, T stage, and N stage were significant predictors of OS **(Fig. 5A)**. In the multivariate Cox regression analysis, the risk score remained a significant predictor, highlighting its independent prognostic value **(Fig. 5B)**. A nomogram was developed to predict 1-, 2-, and 3-year OS probabilities, integrating age, grade, T stage, N stage, and risk score **(Fig. 5C)**. The calibration curves for 1-, 2-, and 3-year OS demonstrated good agreement between the predicted and observed outcomes, indicating the model's accuracy **(Fig. 5D-F)**. The time-dependent ROC analysis showed ideal predictive accuracy with AUC values of 0.81, 0.67, and 0.71 for 1-, 2-, and 3-year survival, respectively **(Fig. 5G)**. These findings suggest that the risk model is a potential independent predictor of OS.

### The correlation between immune cell infiltration and risk score

**Figure 6:**
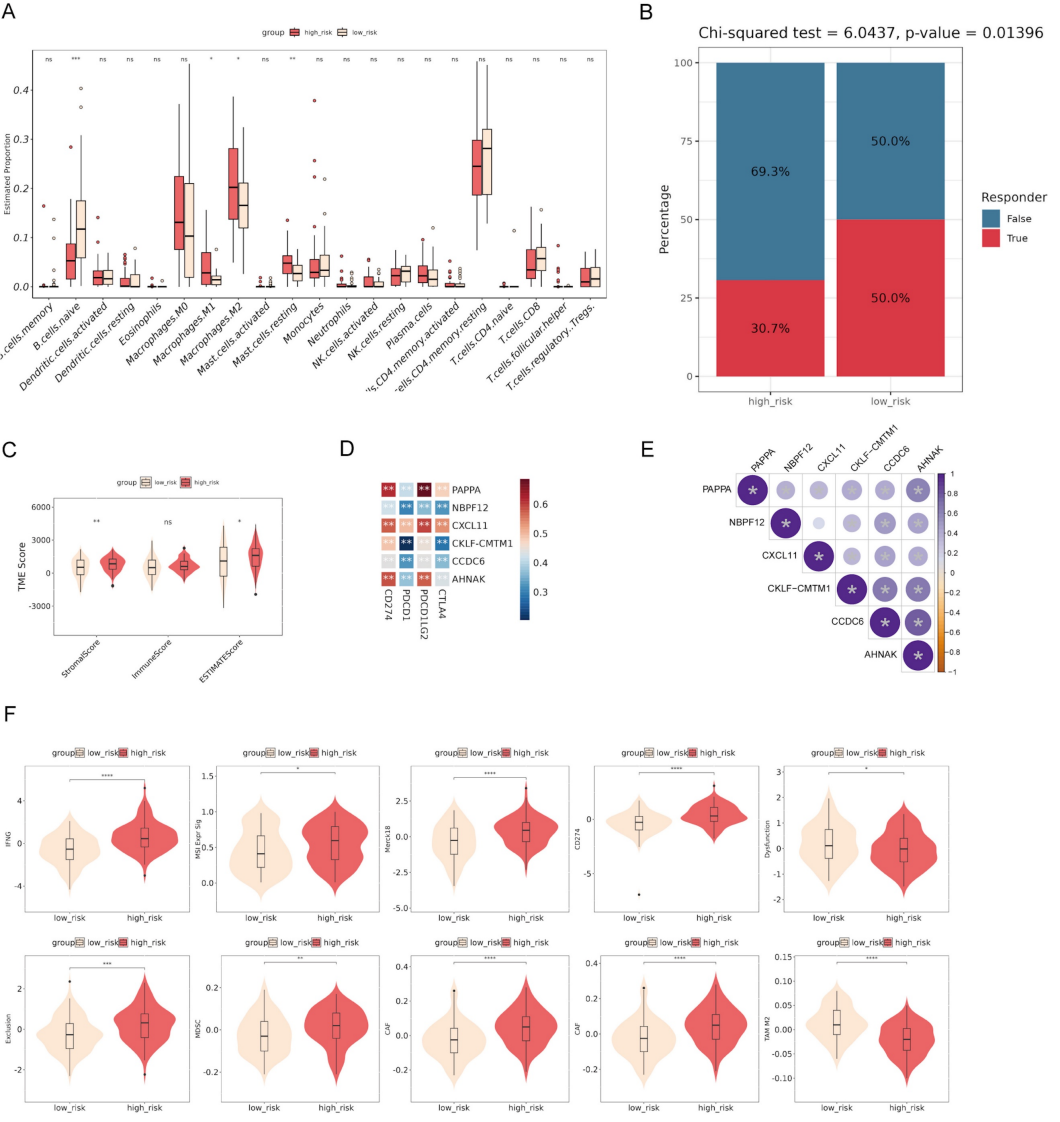
** Correlation between risk score and immune infiltration.** (A) The comparison of immune cell infiltration between high-risk and low-risk groups shows significant differences in the proportions of various immune cells. (B) The Chi-squared test reveals that high-risk patients have a lower response rate to therapy compared to low-risk patients. (C) Analysis of tumor microenvironment (TME) scores demonstrates that high-risk patients have significantly higher StromalScore and ESTIMATEScore. (D) Correlation analysis identifies significant associations between the expression of model genes and immune checkpoints. (E) The bubble plot shows significant correlations among the model genes. (F) Comparisons of various immune-related scores between high-risk and low-risk groups show significant differences. ^*^p < 0.05, ^***^p < 0.001, ^****^p < 0.0001.

To investigate the immune microenvironment and the clinical relevance of the risk model, we performed various analyses. The comparison of immune cell infiltration between high-risk and low-risk groups revealed significant differences in the proportions of naive B cells, M1 macrophages, M2 macrophages, and resting mast cells, suggesting an altered tumor microenvironment (TME) in high-risk patients **(Fig. 6A)**. The Chi-squared test showed that high-risk patients had a lower response rate to therapy compared to low-risk patients, suggesting the prognostic value of the risk score **(Fig. 6B)**. The analysis of TME scores demonstrated that high-risk patients had significantly higher StromalScore and ESTIMATEScore, but no significant difference in ImmuneScore, suggesting a more pronounced stromal component within the tumor **(Fig. 6C)**. Correlation analysis identified significant associations between the expression of the model genes and immune checkpoints **(Fig. 6D)**. The bubble plot showed significant correlations among the model genes **(Fig. 6E)**. Additionally, comparisons of various immune-related scores between high-risk and low-risk groups showed significant differences **(Fig. 6F)**. These findings suggest the potential of the risk model to stratify patients based on their immune microenvironment and may help predict their response to immunotherapy.

### GSEA enrichment analysis and drug sensitivity in patients with different risks

**Figure 7:**
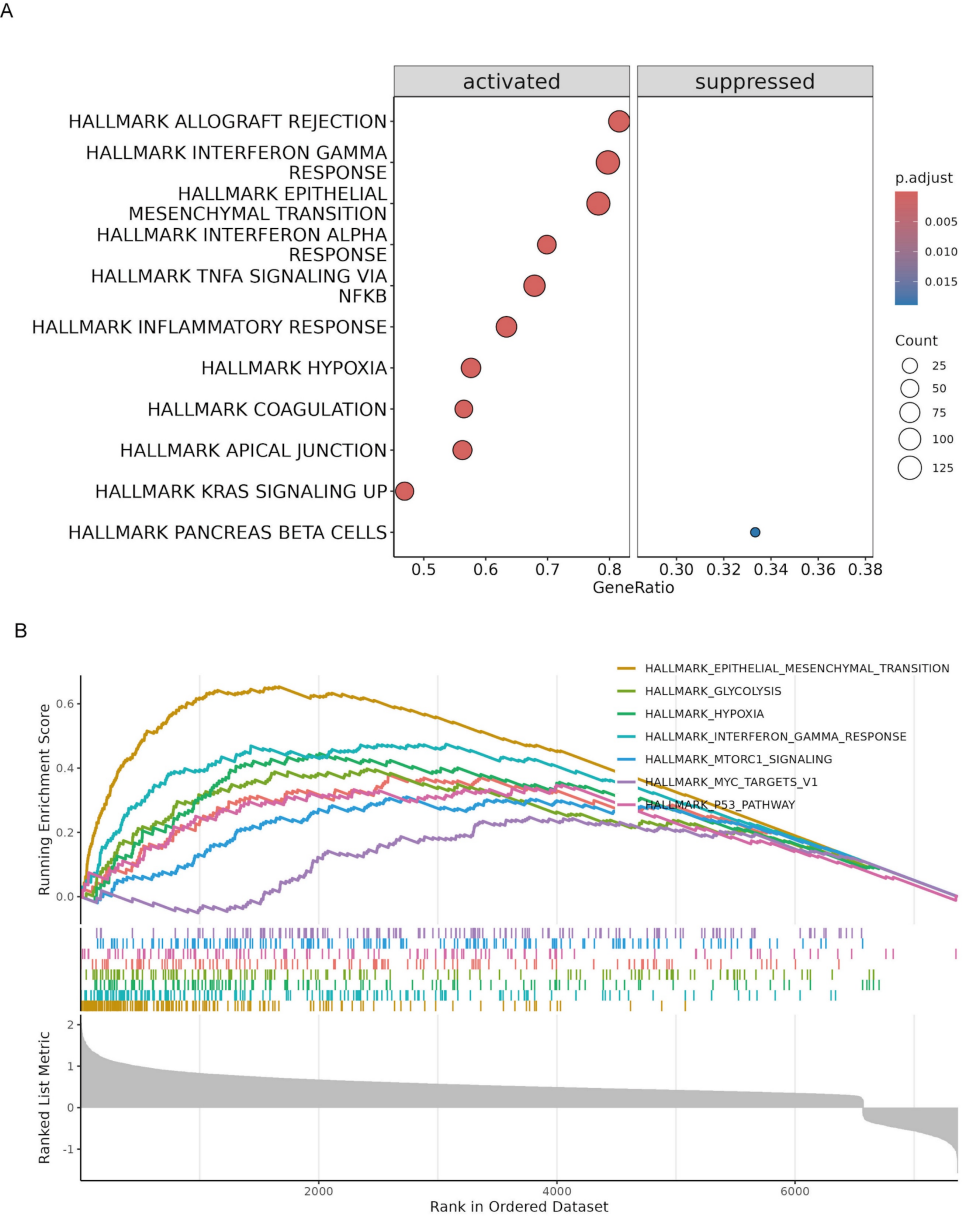
** Gene set enrichment analysis (GSEA) of high-risk PaC patients in the TCGA cohort.** (A) Bubble plot illustrating the significantly enriched hallmark pathways in high-risk patients. The pathways are categorized into activated (left) and suppressed (right) groups. (B) Enrichment plots for specific hallmark pathways significantly enriched in high-risk patients. The x-axis represents the rank in the ordered dataset, and the y-axis shows the running enrichment score.

To further elucidate the biological pathways associated with the risk model, we conducted GSEA in the TCGA cohort. The analysis identified several hallmark pathways that were significantly enriched in high-risk patients. Specifically, pathways such as allograft rejection, interferon gamma response, epithelial-mesenchymal transition, interferon alpha response, TNFA signaling via NFκB, inflammatory response, hypoxia, coagulation, apical junction, and KRAS signaling were activated in high-risk patients. In contrast, the pancreatic beta cells pathway was suppressed **(Fig. 7A)**. The enrichment plots for specific pathways illustrated the distribution of pathway-related genes across the ranked list of all genes **(Fig. 7B)**. All GSEA results are summarized in **Table S6**. This enrichment analysis provides insights into the molecular mechanisms underlying the poorer prognosis observed in high-risk patients, highlighting potential therapeutic targets for intervention.

To explore the potential therapeutic implications of the risk model, we conducted a drug sensitivity analysis **(Table S7)**. High-risk patients showed higher sensitivity to dasatinib and staurosporine, as indicated by lower drug response scores **(Fig. S5A)**. These two drugs showed strong negative correlations with the risk score **(Fig. S5B)**, suggesting increased effectiveness in high-risk patients.

**Figure 8:**
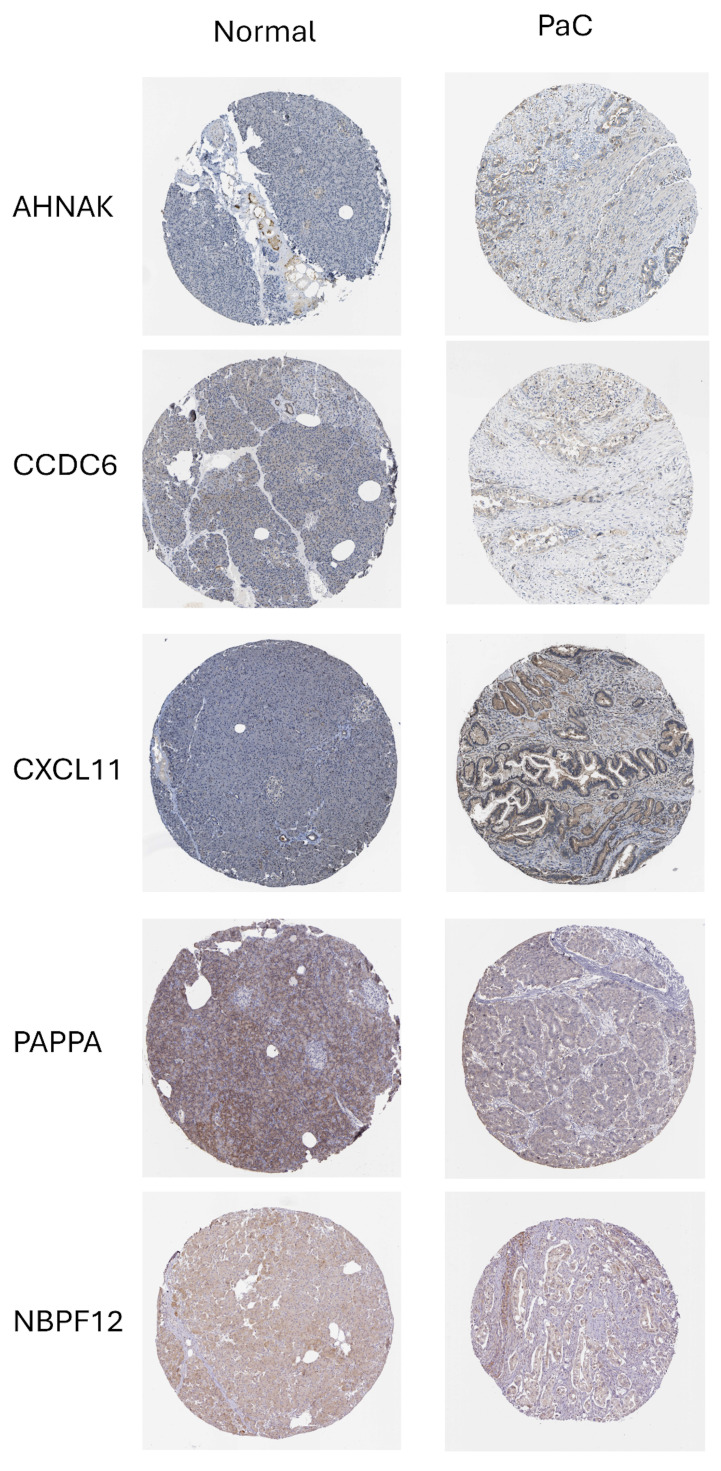
** Protein expression of prognostic model genes in pancreatic tissues.** Representative immunohistochemical staining images of prognostic model genes in normal pancreatic tissue and PaC were obtained from the Human Protein Atlas. PAPPA, NBPF12, CXCL11, CCDC6, and AHNAK show detectable protein expression with heterogeneous staining patterns across samples. CKLF-CMTM1 is not shown due to its read-through nature and the lack of a curated protein annotation in the database.

To support the biological plausibility of the prognostic model at the protein level, we examined immunohistochemical expression patterns using data from the Human Protein Atlas (HPA). Representative images demonstrated detectable and heterogeneous protein expression of PAPPA, NBPF12, CXCL11, CCDC6, and AHNAK in normal pancreatic tissue and PaC **(Fig. 8)**. CKLF-CMTM1 could not be evaluated at the protein level due to its read-through nature and the absence of a curated protein annotation in the database. Although immunohistochemistry does not provide a quantitative assessment of expression changes, these observations indicate that the majority of model genes are translated and expressed in relevant pancreatic tissues, providing supportive protein-level evidence consistent with the RNA-based prognostic model.

## Discussion

The study identified 12 DeMRGs involved in PaC through differential expression analysis. Consensus clustering defined three distinct PaC subtypes based on the DeMRG expression profile, with notable differences in immune cell abundance and prognostic outcomes. A risk model based on six genes among DEGs in PaC subtypes demonstrated prognostic value, stratifying patients into high- and low-risk groups with significant differences in OS and drug sensitivity. Clinically, the risk model may help with patient stratification, prognostic assessment, and identification of potential therapeutic targets, thereby enhancing personalized treatment approaches in PaC.

The six-gene model, comprising PAPPA, NBPF12, CXCL11, CKLF-CMTM1, CCDC6, and AHNAK, along with a nomogram integrating clinical variables, effectively stratified patients into high- and low-risk groups, demonstrating significant prognostic value and differences in OS and drug sensitivity in PaC. NBPF12 is the only gene in the model that is downregulated in PaC tissues compared to adjacent normal tissues. NBPF12 encodes a member of the neuroblastoma breakpoint family, which plays a crucial role in neuroblastoma development and human evolution and is regulated by NF-κB^20^. Although no studies have directly demonstrated a role for NBPF12 in pancreatic biology or mitophagy, recurrent NBPF12 mutations have been reported in multiple cancer genomics studies^21^-^23^, and functional work on other NBPF family members has shown their involvement in cell proliferation via NF-κB signaling^24^. Data from the Human Protein Atlas further confirm that NBPF12 is downregulated in PaC tissues compared to normal tissues (https://v19.proteinatlas.org/ENSG00000268043-NBPF12/pathology/pancreatic+cancer). Our bioinformatic analysis therefore represents a novel finding, identifying NBPF12 for the first time as a prognostic biomarker in PaC and revealing its negative correlation with immune checkpoint expression, suggesting a potential role in immune modulation.

PAPPA (pregnancy-associated plasma protein-A) is a zinc metalloproteinase that enhances IGF action by cleaving inhibitory IGF-binding proteins, increasing IGF availability for cell processes. It promotes tumor growth, invasion, and metastasis by augmenting IGF receptor signaling, contributing to cell proliferation, migration, and survival. PAPPA is implicated in breast, ovarian, and lung cancers, as well as Ewing sarcoma, making it a potential therapeutic target^25^-^28^. Although the direct role of PAPPA in PaC remains unclear, IGF signaling is known to play a significant role. IGF-1 and IGF-2 secreted by tumor-associated macrophages and myofibroblasts contribute to chemoresistance in PaC by activating insulin/IGF receptors on cancer cells. Inhibiting IGF-1/IGF-1R alongside chemotherapy could enhance treatment efficacy^29^, ^30^. In addition, IGF-1 signaling is crucial for sustaining cancer cell viability by stimulating mitochondrial biogenesis and mitophagy through the induction of BNIP3, thereby influencing therapy responses and cancer phenotype evolution^31^. These findings suggest that the prognostic value of PAPPA found in our study may stem from its role in enhancing IGF receptor signaling, which contributes to chemoresistance, cancer cell viability, mitochondrial biogenesis, and mitophagy.

CXCL11 encodes a chemokine, and functional studies have shown that its overexpression promotes, while siRNA-mediated knockdown suppresses, proliferation, migration, invasion, and epithelial-mesenchymal transition (EMT) in PaC cells via the YY1/miR-548t-5p axis^32^. Additionally, CXCL11 is significantly overexpressed in the serum of pretreated PaC patients and differentially expressed in response to gemcitabine and erlotinib treatment, indicating its potential as both a diagnostic and predictive biomarker for PaC^33^. Moreover, the positive correlation between CXCL11 and immune checkpoints in our study aligns with previous research. CXCL11 promotes T-cell infiltration into the TME, increasing the presence of cells that express these immune checkpoints^34^. Specifically, CTLA4 and PD-1 (PDCD1) are upregulated in T cells as a counter-regulatory mechanism to prevent overactivation of the immune response. PD-L1 (CD274) and PD-L2 (PDCD1LG2) bind to PD-1, further contributing to immune suppression^35^. This correlation indicates that CXCL11 enhances immune cell recruitment and subsequently the expression of inhibitory signals within the TME, balancing immune activation and suppression. These insights, along with our findings that CXCL11 is a significant component of our prognostic model, suggest that targeting CXCL11 could improve the efficacy of immune checkpoint blockade therapies in PaC.

CCDC6 encodes a coiled-coil domain-containing protein. Recent work has identified CCDC6 as a mitophagy subtype-specific biomarker in PaC, associated with poor prognosis, altered immune infiltration, and variable drug response^8^. In addition, siRNA-mediated knockdown of AHNAK in PaC cells has been shown to reduce proliferation and migration and to reverse EMT, supporting its role in disease progression^36^. CKLF-CMTM1 is a read-through transcript combining the CKLF and CMTM1 genes, producing a fusion protein involved in immune responses and potentially influencing cancer progression^37^​. However, the role of CKLF-CMTM1 in PaC has not been reported, providing a novel avenue for further research into its potential implications in PaC progression and immune modulation.

The risk model stratified PaC patients into high-risk and low-risk groups, with high-risk patients demonstrating significantly worse OS and distinct immune microenvironment characteristics, suggesting its predictive potential for guiding personalized treatment strategies. Additionally, the model identified high-risk patients as more likely to respond to dasatinib and staurosporine, highlighting its therapeutic relevance in predicting drug sensitivity. Dasatinib, a multi-targeted tyrosine kinase inhibitor, has been used in clinical trials for the treatment of various cancers, including PaC^38^. The combination of dasatinib with paclitaxel or gemcitabine significantly enhances the inhibition of cell viability, proliferation, migration, and colony formation in PaC cell lines by targeting p-SRC, p-STAT3, p-AKT, and p-ERK^39^. Staurosporine, a potent kinase inhibitor, is known for its ability to induce apoptosis by inhibiting various kinase pathways and significantly induces apoptosis in PaC cells by activating caspase-9 and downregulating Bcl2 and Bad expression^40^. Our findings underscore the potential of incorporating dasatinib and staurosporine into therapeutic regimens for improving patient outcomes in high-risk PaC cases.

Our findings demonstrate that the developed risk model effectively stratifies PaC patients into high-risk and low-risk groups, offering a promising tool for guiding personalized treatment strategies. However, the study has several limitations. First, it relies on bioinformatic analyses and public database-derived evidence, including qualitative protein expression data, which may not fully capture the biological complexity and interactions present in experimental or clinical contexts. Second, although previous studies have already investigated the functional roles of CXCL11 and AHNAK in PaC using overexpression or knockdown approaches, their specific involvement in mitophagy has not been assessed, and comparable validation is not yet available for CCDC6, PAPPA, NBPF12, and CKLF-CMTM1. Future studies are required to determine whether these genes directly regulate mitophagy in PaC through similar models. Third, although the 12 DeMRGs were sourced from curated mitophagy-related gene sets and showed enrichment in pathways such as mitochondrial autophagy and organelle disassembly, some may primarily reflect broader mitochondrial quality control or stress response mechanisms rather than direct mitophagy regulation. Distinguishing these roles requires further mechanistic work. Fourth, the altered immune infiltration observed between high- and low-risk groups reflects correlation rather than causation. Whether mitophagy-related genes directly drive immune evasion in PaC remains unresolved. Future mechanistic studies are essential to clarify these potential links. Lastly, the findings require validation in larger, independent cohorts and clinical trials, as predictive performance in external cohorts was modest.

## Conclusions

In conclusion, we identified 12 DeMRGs in PaC tissues and defined three distinct PaC subtypes based on these DeMRGs, each exhibiting unique gene expression profiles and immune cell compositions. Our prognostic risk model, incorporating six DEGs among subtypes, demonstrated predictive value for OS, with high-risk patients showing significantly poorer outcomes. We observed notable differences in the TME of high-risk patients and identified potential benefits from treatments like dasatinib and staurosporine. These findings may contribute to more personalized and targeted treatment approaches for high-risk PaC patients. Validation in prospective clinical cohorts is critical to establish their real-world applicability.

## Supplementary Material

Supplementary figures and tables.

## Figures and Tables

**Table 1:** Sample information.

ID	Platform	Sample type	Sample size (n)	Data type
TCGA		Cancer and adjacent normal tissues	177:4 (Case:Ctrl)	RNAseq
GTEx		Normal pancreatic tissues	167	RNAseq
GSE71729	GPL20769	Cancer tissues	125	mRNA array
GSE21501	GPL4133	Cancer tissues	102	mRNA array

## Abbreviations

PaC: Pancreatic cancer
DEGs: Differentially expressed genes
MRGs: Mitophagy-related genes
TCGA: The Cancer Genome Atlas
GTEx: Genotype-Tissue Expression
OS: overall survival
TME: tumor microenvironment
