# Supplementary material for: Molecular Subtyping and Prognostic Prediction in Pancreatic Cancer Based on Mitophagy-Related Genes
Source: Int J Med Sci. 2026 Jan 14;23(2):620–35. doi: 10.7150/ijms.121350 (PMC12825130; doi:10.7150/ijms.121350)
Supplement: Supplementary file 1 — Supplementary figures and tables. [file ijmsv23p0620s1.zip › supplementary legends.pdf]

Legends of Supplementary material

Figure S1. Relationship between clusters, risk scores, and survival status in the TCGA Cohort. (A) t-SNE plot illustrating the separation of the three clusters. (B) Boxplot comparing the risk scores between different clusters. Cluster 3 shows significantly higher risk scores than Cluster 1 and Cluster 2. $^{*}p < 0.05$, $^{**}p < 0.01$. (C) Sankey diagram showing the distribution of patients from each cluster into high-risk and low-risk groups and their corresponding survival status. High-risk patients are depicted in red and low-risk patients in blue, with survival outcomes (alive or dead) represented on the right.

Figure S2. Validation of the prognostic value of the risk model in external PaC cohorts. (A, B) Risk factor distribution and survival status plots for GSE21501 (A) and GSE71729 (B). (C, D) Kaplan-Meier survival curves for GSE21501 (C) and GSE71729 (D). (E, F) Time-dependent ROC curves for GSE21501 (E) and GSE71729 (F) at 1 year, 2 years, and 3 years. AUC values are indicated.

Figure S3. Survival analysis stratified by clinical and demographic subgroups. (A–H) Kaplan-Meier survival curves comparing OS between high-risk (red) and low-risk (gray) groups across various clinical and demographic subgroups. The subgroups include (A) Patients aged ≤ 65 years. (B) Patients aged > 65 years. (C) Female patients. (D) Male patients. (E) Patients with well-differentiated tumors (G1-G2). (F) Patients with poorly-differentiated tumors (G3-G4). (G) Patients with early-stage disease (Stage I-II). (H) Patients with advanced-stage disease (Stage III-IV).

Figure S4. Survival analysis and risk score distribution by tumor stage and lymph node status. (A–F) Kaplan-Meier survival curves comparing OS between high-risk (red) and low-risk (gray) groups stratified by (A) Tumor stage T1-T2. (B) Tumor stage T3-T4. (C) Absence of metastasis (M0). (D) Presence of metastasis (M1). (E) Absence

of lymph node involvement (N0). (F) Presence of lymph node involvement (N1-N3). (G) Box plots illustrating the distribution of risk scores across different tumor grades and stages. Left plot: Risk scores by tumor grade (G1-G2 vs. G3-G4). Middle plot: Risk scores by tumor stage (T1-T2 vs. T3-T4). Right plot: Risk scores by lymph node status (N0 vs. N1-N3).

Figure S5. Drug sensitivity prediction in patients across different risk groups. (A) Comparison of predicted sensitivity scores (PreScores) between high-risk (yellow) and low-risk (blue) patients for various drugs. (B) Correlation analysis between the risk score and drug sensitivity reveals significant associations. The size of the circles represents the strength of the correlation (cor1), with larger circles indicating stronger correlations. Drugs with p-values < 0.05 are shown.

Table S1 DEGs in PaC tissues compared to normal controls.

Table S2 GO functional enrichment analysis of DeMRGs.

Table S3 GO functional enrichment analysis of DEGs among PaC subtypes.

Table S4 KEGG pathway enrichment analysis of DEGs among PaC Subtypes.

Table S5 Univariate COX analysis of potential prognostic genes among DEGs in the TCGA cohort.

Table S6 Results of hallmark gene set enrichment analysis (GSEA).

Table S7 Results of drug prediction analysis using oncoPredict.
