# Supplementary figures and images for: Molecular Subtyping and Prognostic Prediction in Pancreatic Cancer Based on Mitophagy-Related Genes
Source: Int J Med Sci. 2026 Jan 14;23(2):620–35. doi: 10.7150/ijms.121350 (PMC12825130; doi:10.7150/ijms.121350)

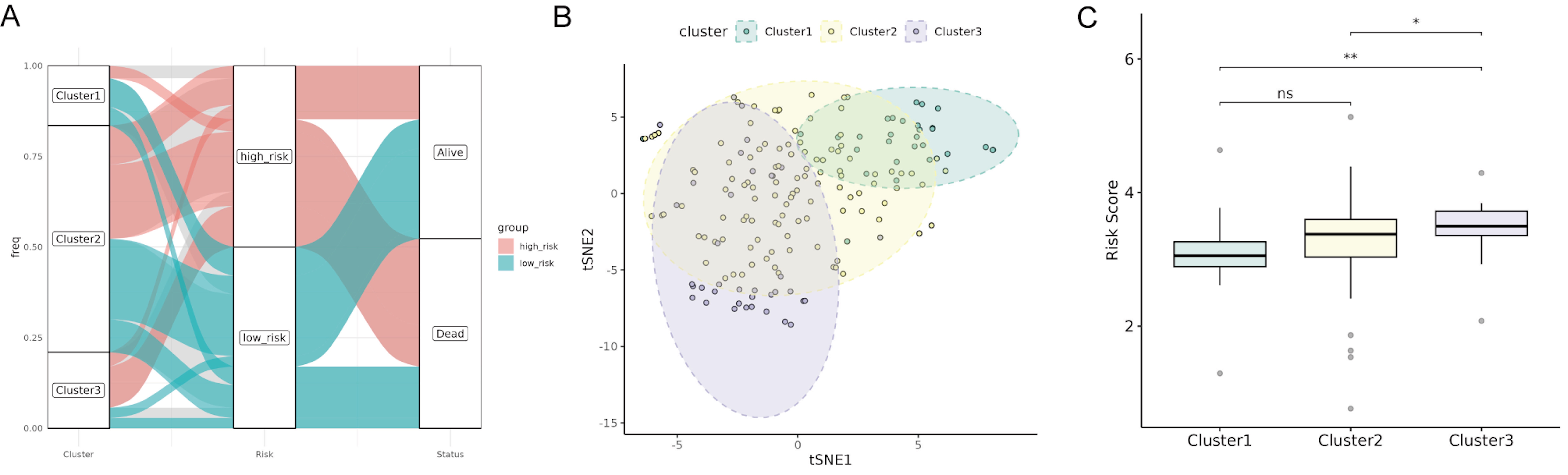

Supplement: Supplementary file 1 — Supplementary figures and tables. [file ijmsv23p0620s1.zip › Supplementary figure/Figure S1.jpg]

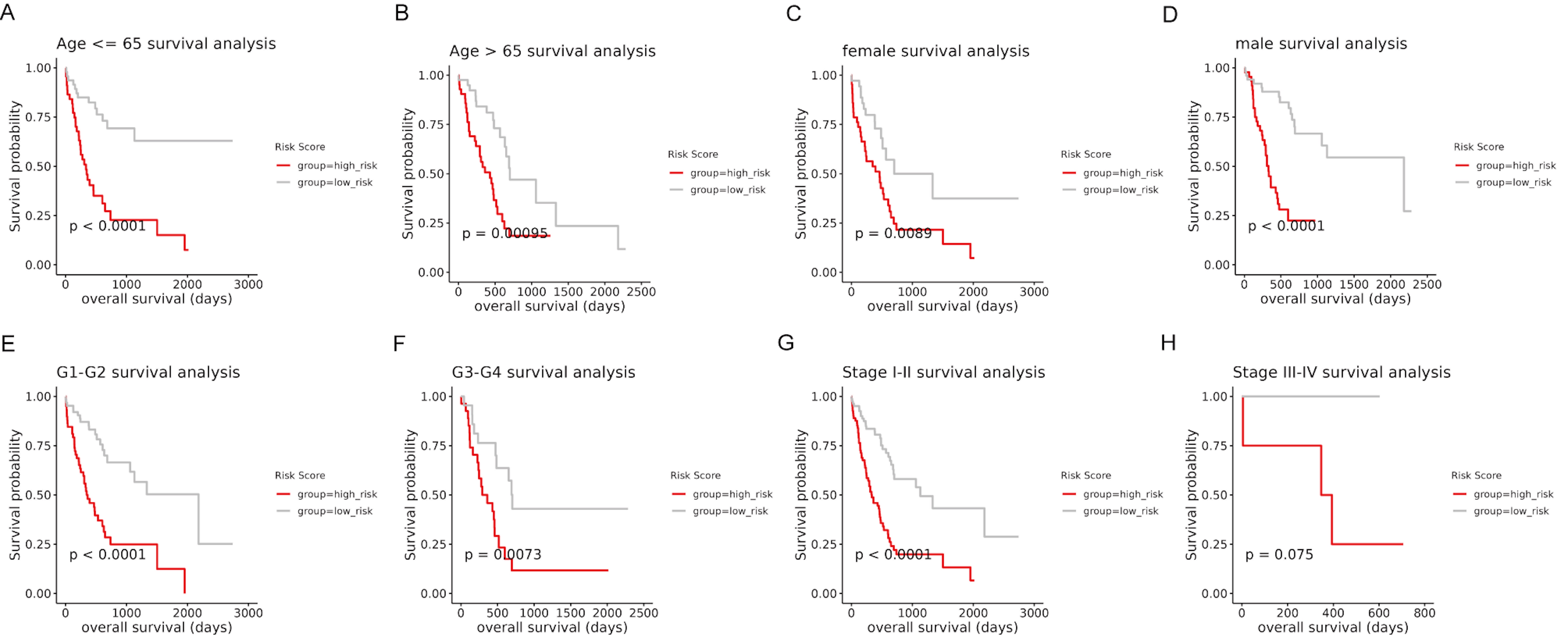

Supplement: Supplementary file 1 — Supplementary figures and tables. [file ijmsv23p0620s1.zip › Supplementary figure/Figure S3.jpg]

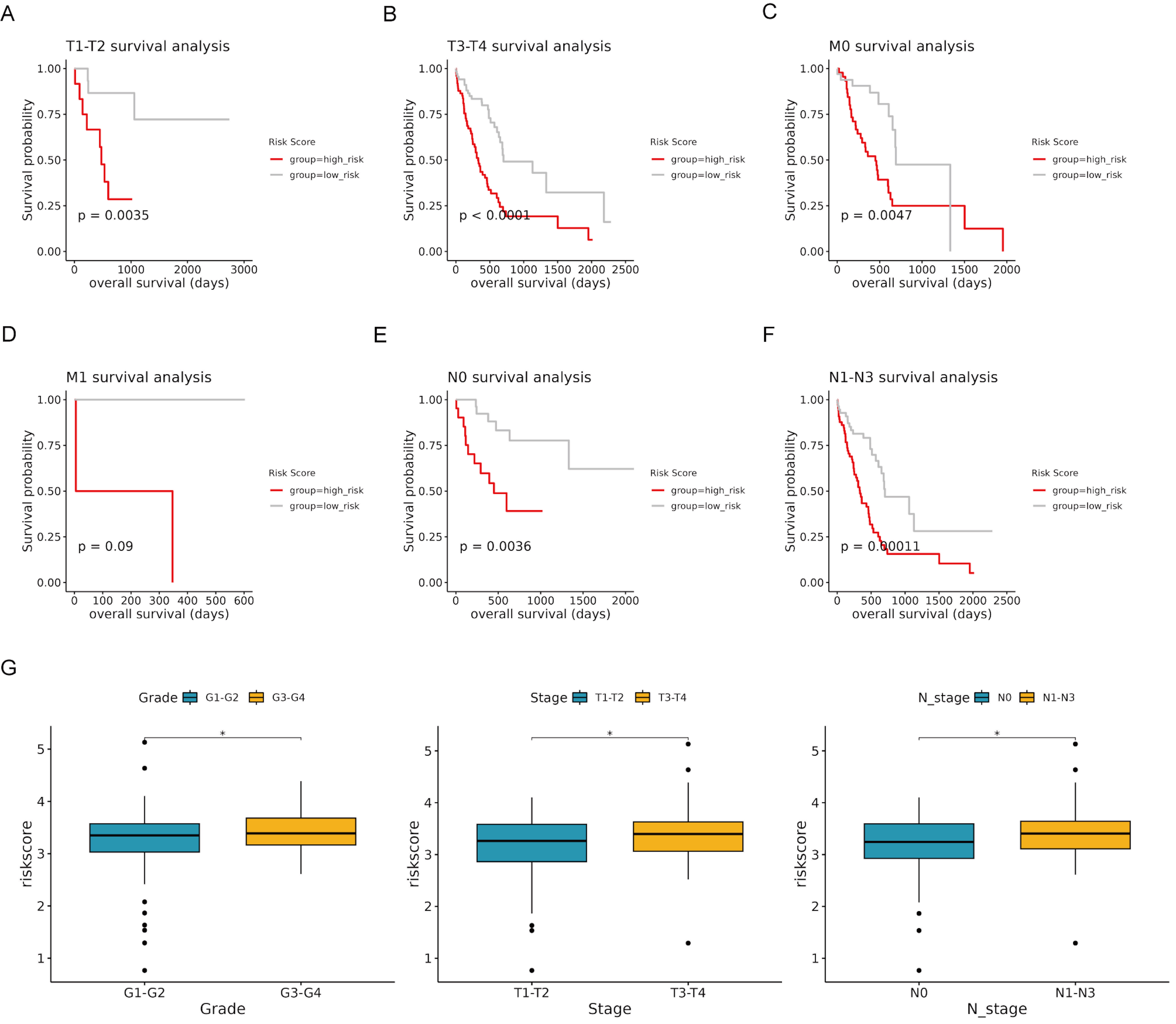

Supplement: Supplementary file 1 — Supplementary figures and tables. [file ijmsv23p0620s1.zip › Supplementary figure/Figure S4.jpg]

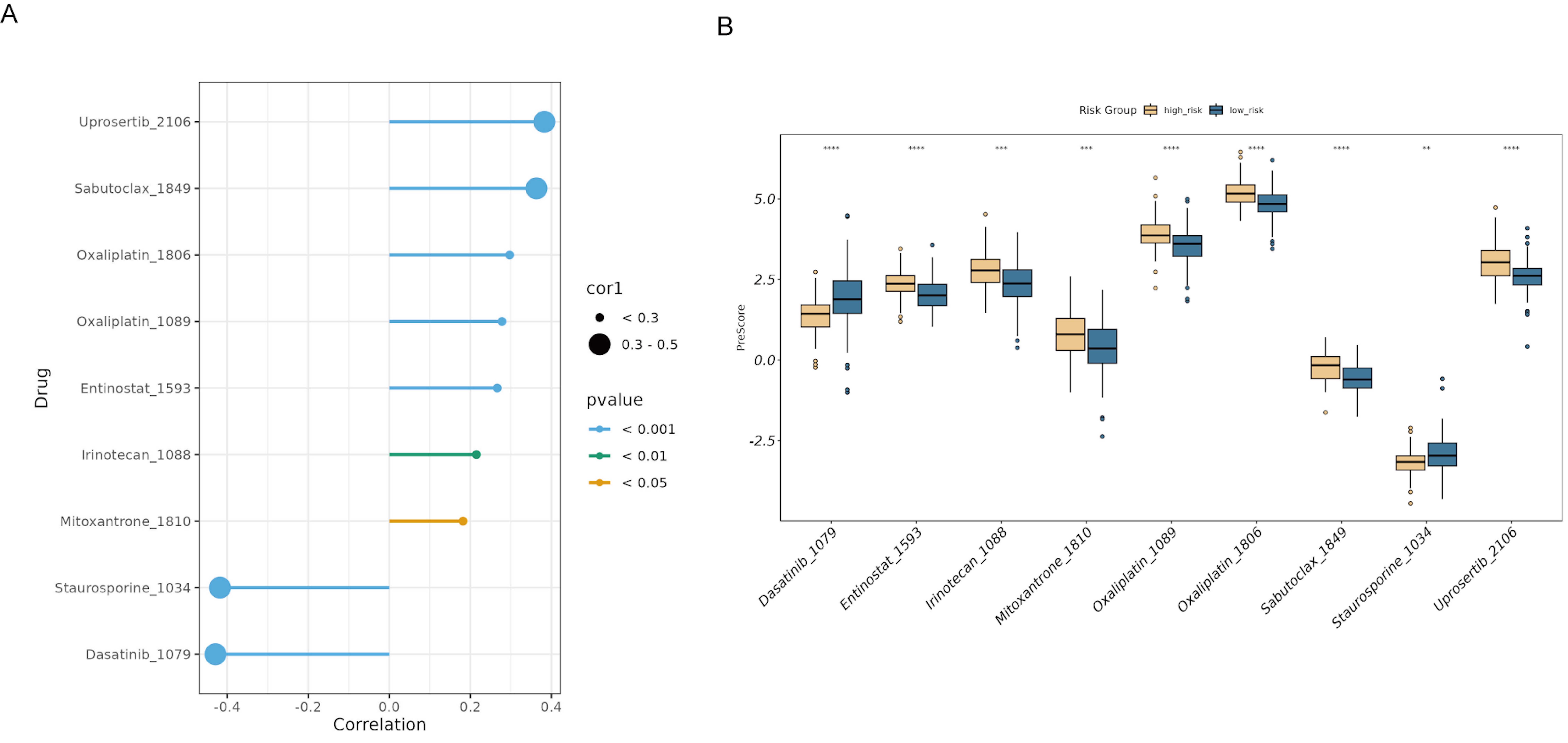

Supplement: Supplementary file 1 — Supplementary figures and tables. [file ijmsv23p0620s1.zip › Supplementary figure/Figure S5.jpg]
